# CNS safety at 48-week of switching to ATV/r plus 3TC or two nucleos(t)ides in HIV-suppressed patients on stable ART: the SALT neurocognitive sub-study

**DOI:** 10.7448/IAS.17.4.19656

**Published:** 2014-11-02

**Authors:** Ignacio Pérez Valero, Juan Pasquau, Rafael Rubio, Antonio Ribero, Jose Santos, Jesus Sanz, Ana Mariño, Manel Crespo, Jose Hernandez, Jose Antonio Iribarren, Felix Gutierrez, Alberto Terron, Herminia Esteban, Jose Antonio Pérez-Molina

**Affiliations:** 1 Internal Medicine, H.U. La Paz, Madrid, Spain; 2 Internal Medicine, Hospital Virgen de las Nieves, Granada, Spain; 3 Internal Medicine, H.U. Doce de Octubre, Madrid, Spain; 4 Internal Medicine, Hospital Reina Sofia, Cordoba, Spain; 5 Internal Medicine, Hospital Virgen de la Victoria, Malaga, Spain; 6 Internal Medicine, Hospital de Alcala de Henares, Alcala de Henares, Spain; 7 Internal Medicine, Hospital Arquitecto Marcide, Ferrol, Spain; 8 Internal Medicine, Hospital Vall d'Hebron, Barcelona, Spain; 9 Internal Medicine, Hospital San Cecilio, Granada, Spain; 10 Internal Medicine, Hospital de Donostia, San Sebastian, Spain; 11 Internal Medicine, Hospital de Elche, Elche, Spain; 12 Internal Medicine, Hospital de Jerez, Jerez, Spain; 13 Fundación SEIMC-GESIDA, Madrid, Spain; 14 Infectious Diseases, Hospital Ramón y Cajal, Madrid, Spain

## Abstract

**Introduction:**

Due to their low CNS penetrance, there are concerns about the capacity of non-conventional PI-based ART (monotherapy and dual therapies) to preserve neurocognitive performance (NP).

**Methods:**

We evaluated the NP change of aviremic participants of the SALT clinical trial [1] switching therapy to dual therapy (DT: ATV/r+3TC) or triple therapy (TT: ATV/r+2NRTI) who agreed to perform an NP assessment (NPZ-5) at baseline and W48. Neurocognitive impairment and NP were assessed using AAN-2007 criteria [2] and global deficit scores (GDS) [3]. Neurocognitive change (GDS change: W48 – baseline) and the effect of DT on NP evolution crude and adjusted by significant confounders were determined using ANCOVA.

**Results:**

A total of 158 patients were included (Table 1). They had shorter times because HIV diagnosis, ART initiation and HIV-suppression and their virologic outcome at W48 by snapshot was higher (79.1% vs 72.7%; p=0.04) compared to the 128 patients not included in the sub-study. By AAN-2007 criteria, 51 patients in each ART group (68% vs 63%) were neurocognitively impaired at baseline (p=0.61). Forty-seven patients were not reassessed at W48: 30 lost of follow-up (16 DT-14 TT) and 17 had non-evaluable data (6 DT-11 TT). Patients retested were more likely to be men (78.9% vs 61.4%) and had neurological cofounders (9.6% vs 0%) than patients non-retested. At W48, 3 out of 16 (5.7%) patients on DT and 6 out of 21 (10.5%) on TT who were non-impaired at baseline became impaired (p=0.49) while 10 out of 37 (18.9%) on DT and 7 out of 36 (12.3%) on TT who were neurocognitively impaired at baseline became non-impaired (p=0.44). Mean GDS changes (95% CI) were: Overall −0.2 (−0.3 to −0.04): DT −0.26 (−0.4 to −0.07) and TT −0.08 (−0.2 to 0.07). NP was similar between DT and TT (0.15). This absence of differences was also observed in all cognitive tests. Effect of DT: −0.16 [−0.38 to 0.06]) (r^2^=0.16) on NP evolution was similar to TT (reference), even after adjusting (DT: −0.11 [−0.33 to 0.1], TT: reference) by significant confounders (geographical origin, previous ATV use and CD4 cell count) (r^2^=0.25).

**Conclusions:**

NP stability was observed after 48 weeks of follow up in the majority of patients whether DT or TT was used to maintain HIV-suppression. Incidence rates of NP impairment or NP impairment recovery were also similar between DT and TT.

**Table 1:** Baseline characteristics

	ATV/r + 3TC N = 76 (48.1%)	ATV/r + 2 NRTI N = 82 (51.9%)	p
Male. N (%)	54 (71.1)	63 (76.8)	0.47
Age. Mean (SD)	43.1 (10.3)	41.6 (8.6)	0.32
Immigrants (%)	19 (25%)	18 (22%)	0.09
Years of education. Mean (SD)	13.0 (4.7)	13.6 (5.0)	0.48
AIDS. N (%)	24 (31.6)	22 (26.8)	0.60
Years since HIV detection. Mean (SD)	7.5 (6.2)	7.3 (6.4)	0.57
Years with HIV-RNA < 50 c/mL. Mean (SD)	3.1 (2.9)	3.0 (2.7)	0.92
Nadir CD4 (cells/mm^3^). Median (IQR)	211 (70–325)	193 (130–300)	0.90
Baseline CD4 (cells/mm^3^). Median (IQR)	575 (388–772)	624 (417–825)	0.12
Years of ART. Mean (SD)	3.8 (3.0)	3.6 (2.3)	0.94
Hepatitis C Antibody +. N (%)	16 (21.1)	16 (19.5)	0.85
Aterogenic index (Total CHO/HDL), median (IQR)	3.9 (3.3–4.8)	3.9 (3.3–4.7)	0.76
Presence of neurological comorbidities. N (%)	3 (3.9)	8 (9.8)	0.21
Presence of psychiatric comorbidities. N (%)	36 (47.4)	42 (51.2)	0.64
Presence of cardiovascular comorbidities. N (%)	45 (59.2)	41 (50)	0.27
